# Macrophage-based therapy for intervertebral disc herniation: preclinical proof-of-concept

**DOI:** 10.1038/s41536-023-00309-z

**Published:** 2023-07-10

**Authors:** Cláudia Ribeiro-Machado, Susana G. Santos, Inês A. Amaral, Joana Caldeira, Paulo Pereira, Mário A. Barbosa, Carla Cunha

**Affiliations:** 1 i3S - Instituto de Investigação e Inovação em Saúde, Universidade do Porto, Porto, Portugal; 2 INEB - Instituto Nacional de Engenharia Biomédica, Universidade do Porto, Porto, Portugal; 3 ICBAS - Instituto de Ciências Biomédicas Abel Salazar, Universidade do Porto, Porto, Portugal; 4 Department of Neurosurgery, Centro Hospitalar Universitário São João, Porto, Portugal; 5 Department of Clinical Neurosciences and Mental Health, Faculty of Medicine, University of Porto, Porto, Portugal; 6 Spine Unit, CUF Porto, Porto, Portugal

**Keywords:** Osteoarthritis, Preclinical research, Macrophages, Immunotherapy

## Abstract

Intervertebral disc (IVD) degeneration and herniation is a leading cause of disability globally and a large unmet clinical need. No efficient non-surgical therapy is available, and there is an urgency for minimally invasive therapies capable of restoring tissue function. IVD spontaneous hernia regression following conservative treatment is a clinically relevant phenomenon that has been linked to an inflammatory response. This study establishes the central role of macrophages in IVD spontaneous hernia regression and provides the first preclinical demonstration of a macrophage-based therapy for IVD herniation. A rat model of IVD herniation was used to test complementary experimental setups: (1) macrophage systemic depletion via intravenous administration of clodronate liposomes (Group CLP2w: depletion between 0 and 2 weeks post-lesion; Group CLP6w: depletion between 2 and 6 weeks post-lesion), and (2) administration of bone marrow-derived macrophages into the herniated IVD, 2 weeks post-lesion (Group Mac6w). Herniated animals without treatment were used as controls. The herniated area was quantified by histology in consecutive proteoglycan/collagen IVD sections at 2 and 6 weeks post-lesion. Clodronate-mediated macrophage systemic depletion was confirmed by flow cytometry and resulted in increased hernia sizes. Bone marrow-derived macrophages were successfully administered into rat IVD hernias resulting in a 44% decrease in hernia size. No relevant systemic immune reaction was identified by flow cytometry, cytokine, or proteomic analysis. Furthermore, a possible mechanism for macrophage-induced hernia regression and tissue repair was unveiled through IL4, IL17a, IL18, LIX, and RANTES increase. This study represents the first preclinical proof-of-concept of macrophage-based immunotherapy for IVD herniation.

## Introduction

Intervertebral disc (IVD) herniation is frequently associated with low back pain, is a highly disabling condition, and is a main cause of spinal surgery worldwide^1,2^. The IVD is a unique tissue that lays between vertebrae and warrants the structural stability of the spine. IVD herniation consists of the disruption of the annulus fibrosus (AF), extrusion of the nucleus pulposus (NP), the release of inflammatory mediators, and stimulation of spinal nerve root fibers, leading to inflammation and pain. Symptoms originating from IVD herniation may subside without surgical treatment, and in some of these patients, this is accompanied by a reduction of the size of IVD herniation, as evident by magnetic resonance imaging (MRI). This phenomenon is known as spontaneous hernia regression. Clinical evidence shows that the regression may be partial or complete, with larger and extruded hernias being more prone to resorption and occurring in 15–43% of the patients^3^. The first clinical approach to IVD herniation is nonsurgical, including physical therapy, nonsteroidal anti-inflammatory drugs, analgesics, and/or corticosteroid injections^2^. However, this implies long periods of treatment and great uncertainty in the outcome. Surgical interventions such as microdiscectomy provide faster pain relief but do not restore tissue function, entail a risk of recurrence and need for revision surgery, and may have associated complications, such as neurological injury^4^. The decision on conservative versus surgical management of IVD herniation is mostly patient-clinician dependent. This is because the mechanisms behind spontaneous hernia regression remain poorly elucidated, resulting in high uncertainty in the prognosis. As so, understanding IVD hernia regression represents a major challenge in spine care.

The main hypothesis for the mechanism underlying spontaneous IVD hernia regression is that exposure of the herniated disc to the epidural vascular supply through the ruptured posterior longitudinal ligament activates immune cells that initiate a cascade of inflammation, angiogenesis, and extracellular matrix (ECM) remodeling reviewed in^5^. Macrophage infiltration has been identified in herniated IVD histological samples and especially in the sequestration subtype, rather than the subligamentous one, which is in accordance with clinical evidence showing that sequestered hernias are more likely to regress^6^. Using a rat IVD herniation model^7^, in two independent experiments, we have consistently observed the presence of CD68+ macrophages in the hernias and demonstrated that the percentage of macrophages is proportional to the hernia size^7,8^. Macrophages are indeed good candidates to explain hernia regression. They are capable of phagocytizing the herniated tissue and also of secreting enzymes that degrade ECM components^9^. We have also recently shown in bovine IVD organ culture that macrophages promote tissue remodeling by downregulating ECM genes in the IVD^10^. A recent observational study using samples collected from patients further supports the role of macrophages in IVD hernia regression^11^.

Macrophages present in the hernia most likely result from blood monocyte recruitment^12^. What regulates the fate of these cells and to what extent they can assume the identity and function of resident macrophages is still unclear. Macrophages are known to promote homeostasis, not only as phagocytes but also through trophic, regulatory, and repair functions. These highly plastic cells can display a range of phenotypes regulated by their microenvironment (including inflammatory cytokines and ECM composition), which span from the classical pro-inflammatory M1 to the alternatively activated anti-inflammatory M2 phenotype^13^. It is possible that the M1 phenotype may be more associated with increased IVD degeneration^14^, but the detailed macrophage profile within the IVD remains undiscovered, and a direct demonstration of macrophage role in IVD herniation is still missing.

Finding effective management and treatment for IVD herniation is clinically and socially relevant, representing an unmet medical need as well as a scientific challenge. This study demonstrates that macrophages are essential for IVD hernia regression and explores the potential of macrophages for restoring IVD homeostasis using a revolutionary concept towards hernia resolution, which may be relevant to the clinical practice, pinpointing its presumptive mechanism of action. Ultimately, such therapy may improve human lifelong health and well-being and result in an outstanding innovation in a field central to low back pain.

## Results

**Fig. 1:**
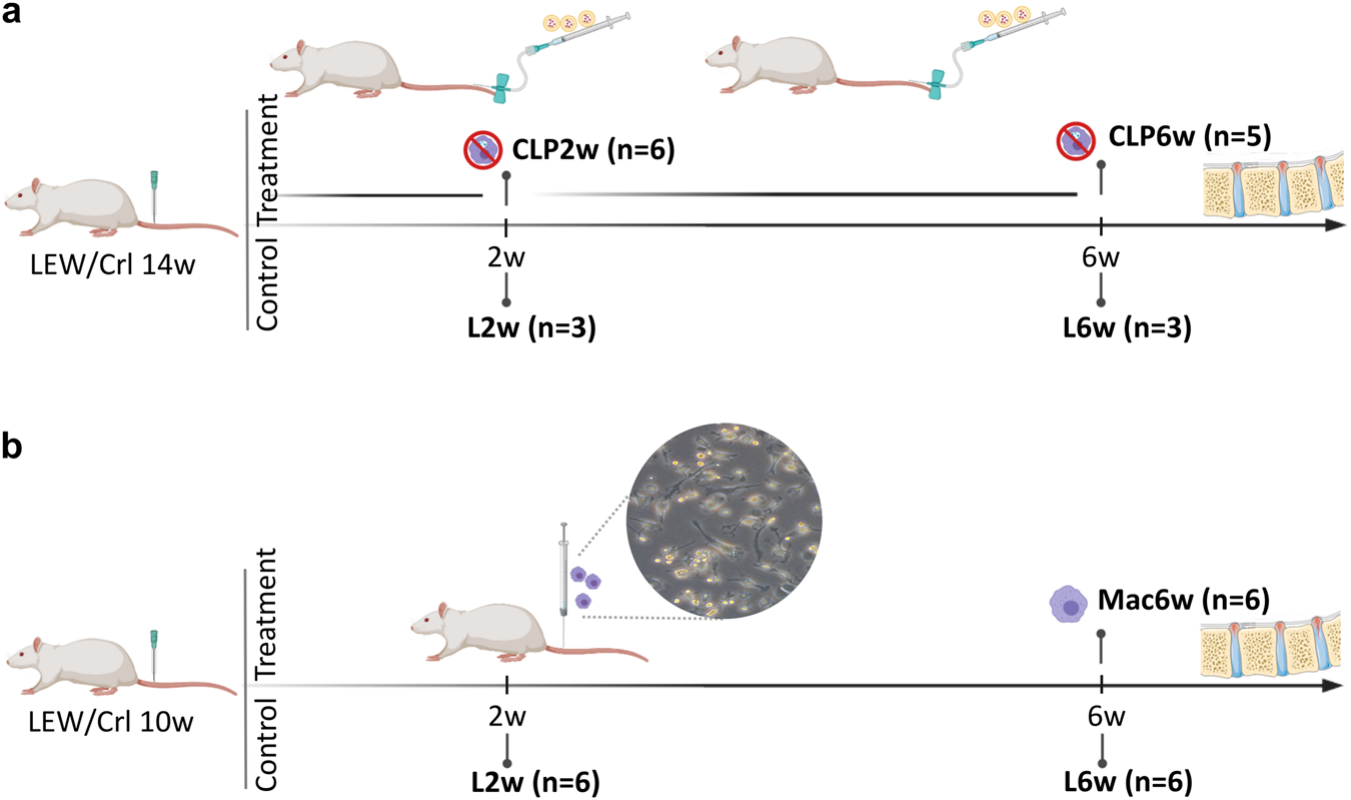
Experimental setup. **a** Macrophage systemic depletion for impairment of IVD hernia regression. Multiple clodronate liposome injections were administered to one group of animals between the lesion and 2 weeks post-lesion (CLP2w, *n* = 6) and to another group of animals between 2 and 6 weeks post-lesion (CLP6w, *n* = 5). Control groups consisted of lesioned animals sacrificed at 2w (L2w, *n* = 3) and 6w (L6w, *n* = 3) post-lesion, without clodronate liposomes administration. **b** Macrophage local delivery for IVD hernia regression. One group of animals received macrophage administration at 2 weeks post-lesion and was sacrificed at 6 weeks post-lesion (Group Mac6w, *n* = 6). Control groups consisted of lesioned animals sacrificed at 2w (L2w, *n* = 6) and 6w (L6w, *n* = 6) post-lesion, without macrophage administration. Inset: monocyte-derived macrophages isolated from the rat bone marrow.

This proof-of-concept study aimed to demonstrate the role of macrophages in IVD hernia regression in vivo. A rat IVD herniation model was used, in which the progress of IVD herniation is well characterized^7^. Complementary loss of function and gain of function experimental setups were followed. In the first approach, macrophages were systemically depleted to demonstrate their role in IVD hernia regression (Fig. 1a), and in the second approach, bone-marrow-derived macrophages were adoptively transferred into the hernia, as an in vivo validation of a macrophage-based therapy for IVD hernia regression (Fig. 1b).

### Macrophage systemic depletion impairs IVD hernia regression

To demonstrate the contribution of macrophages to hernia regression, they were systemically depleted via intravenous administration of commercially available liposomes loaded with clodronate (dichloromethylene-bisphosphonate, Liposoma), a drug that selectively induces apoptosis of macrophages upon phagocytosis^15,16^. Macrophage depletion is expected to occur at around 24 h after intravenous administration, and circulating monocyte depletion is reported to peak at 18 h after administration^17^. However, repopulating monocytes can be recruited from the bone marrow within a relatively short time, so repeated administrations were performed for a more prolonged macrophage and monocyte depletion^16,18^. Two transiently depleted groups were analyzed to understand the macrophage contribution along the progression of hernia formation and regression. The administration schedule was intravenous injections every 3 days starting from the day of the lesion until 2 weeks post-lesion for one group of animals (CLP2w) and every 5 days starting from 2 weeks post-lesion until 6 weeks post-lesion for a second group of animals (CLP6w). The animals were sacrificed at 6 weeks post-lesion. Control groups consisted of lesioned animals sacrificed at 2 (L2w) and 6 weeks (L6w) post-lesion, without clodronate liposomes (CLPs) administration (Fig. 1a).

**Fig. 2:**
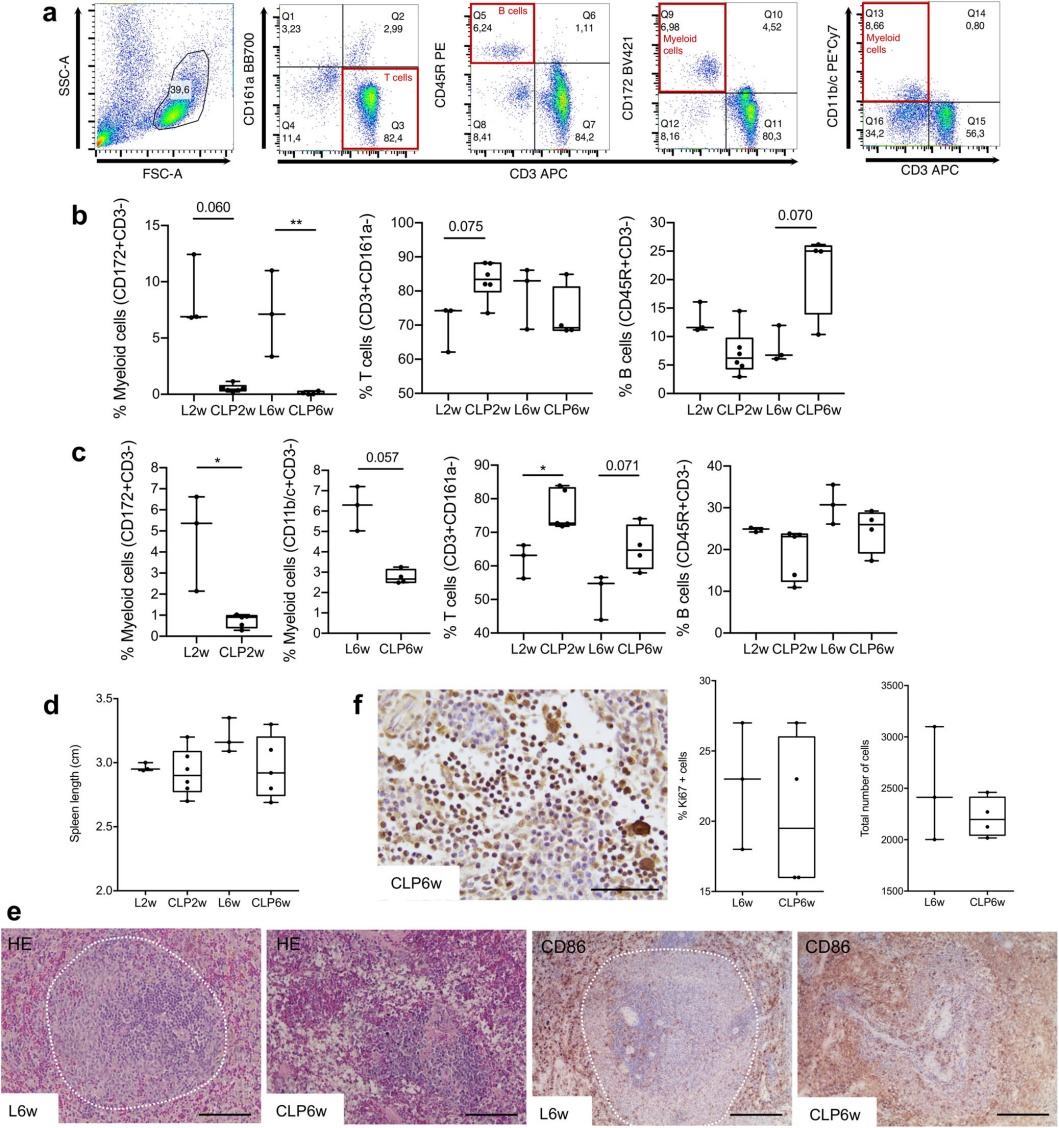
Systemic immune response to macrophage systemic depletion. **a** Representative plots of the flow cytometry gating strategy to identify T-, B-, and myeloid cell populations in peripheral blood and spleen. In the spleen, either CD172 or CD11b/c surface markers were used, as indicated in (**c**). **b** Peripheral blood mononuclear cells immunophenotyping by flow cytometry. **c** Spleen cells immunophenotyping by flow cytometry. **d** Quantification of spleen length. **e** Hematoxylin and Eosin (HE) and immunohistochemistry for CD68+ cells, showing alterations in the spleen tissue architecture when macrophages are depleted. Dashed lines outline the while pulp. Scale bars: 200 µm. **f** Immunohistochemistry for Ki67+ proliferating cells and the total number of cells in the lymph nodes. Scale bar: 50 µm. Results are presented as median ± interquartile range (IQR) in box and whiskers plots, and statistical analysis used the non-parametric Kruskal–Wallis test, followed by Dunn’s multiple comparison test, except for (**f**) in which the Mann–Whitney test was used. *p < 0.05, **p < 0.01.

For confirmation of systemic macrophage depletion by liposomal clodronate treatment, flow cytometry was performed for CD172+ and CD11b/c+ cells in the blood and spleen (Fig. 2a). In the blood, we found a statistically significant decrease in myeloid populations in the CLP2w and CLP6w groups, demonstrating a complete depletion of the macrophage populations (Fig. 2b). Concomitant increases in the proportions of T and B cells were found for the groups CLP2w and CLP6w, respectively, although not reaching statistical significance. In the spleen, a significant decrease in myeloid populations was found for CLP2w (*p* < 0.05), and a decrease was also found for CLP6w (*p* = 0.057). Similarly to the blood, there was an increase in T cell proportions in the group CLP2w (*p* < 0.05) and no statistically significant difference in B cells (Fig. 2c). Although there seems to be a decrease in spleen length for both CLP2w and CLP6w depleted groups, no statistically significant differences were found (Fig. 2d). However, histologically, we found deep alterations in the spleen tissue architecture, which loses the white pulp organization in the depleted groups (Fig. 2e). These results confirm the efficiency of monocytes and macrophage depletion treatment. No alterations were found in proliferating or total number of cells in the lymph nodes between the L6w and CLP6w groups (Fig. 2f).

**Fig. 3:**
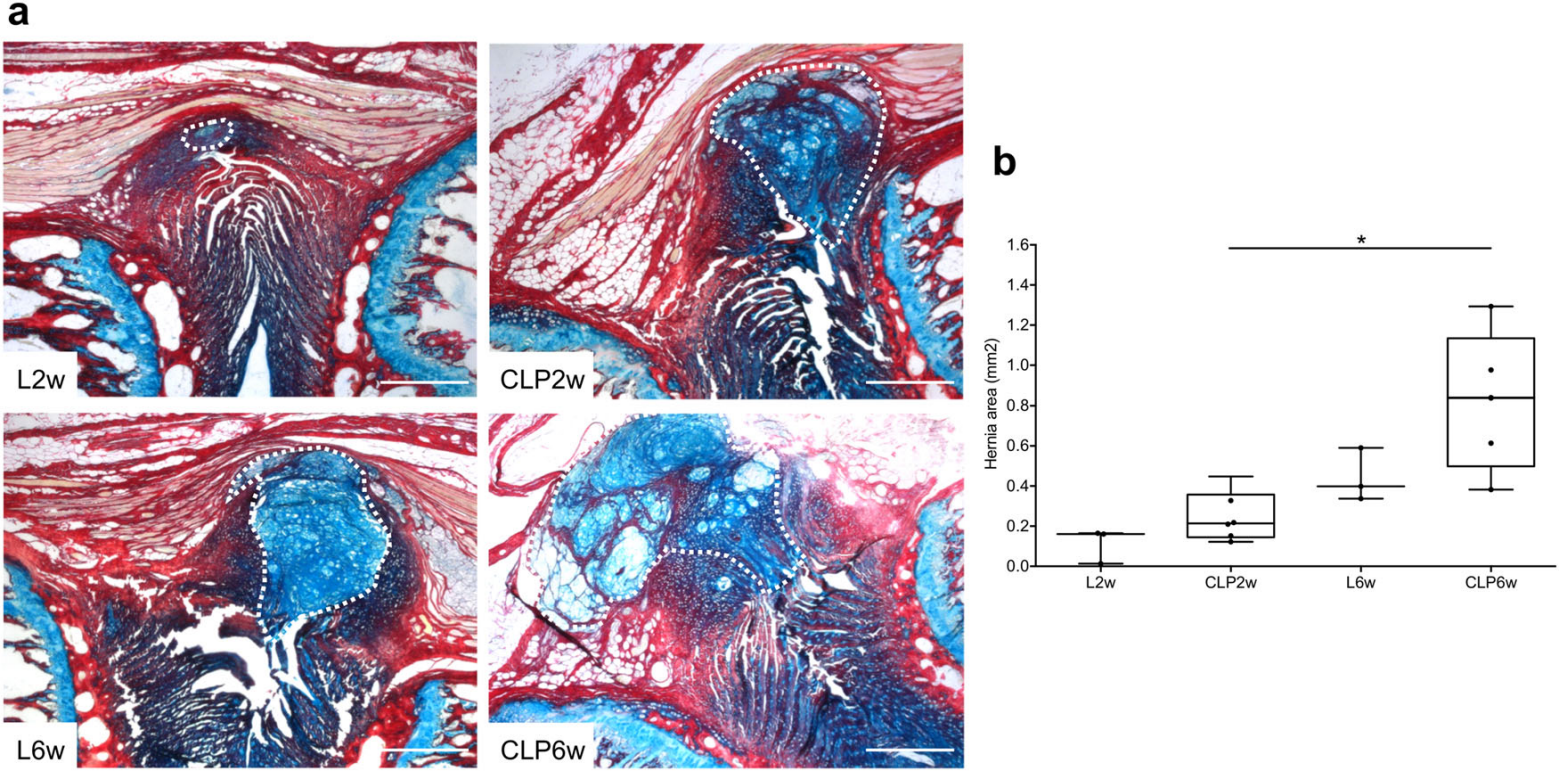
Hernia histopathological analysis at 2 and 6 weeks post-injury and after macrophage depletion. **a** Representative images of the hernia stained by Alcian blue (proteoglycans) and Picrosirius red (collagen). The dashed line outlines the hernia. Scale bars: 500 µm. **b** Quantification of average hernia area (mm^2^) across the depth of all sections of each IVD with visible herniation. Results are presented as median ± interquartile range (IQR) in box and whiskers plots, and statistical analysis used the non-parametric Kruskal–Wallis test, followed by Dunn’s multiple comparison test. *p < 0.05.

Having confirmed macrophage depletion, we proceeded to analyze hernia progression over 2 and 6 weeks. Histological analysis of the herniated area was performed throughout the IVD by proteoglycan/collagen staining (Fig. 3a). Results show an increase in hernia size for both CLP2w and CLP6w depleted groups, compared to their control groups, which was especially evident for CLP6w, between 2 and 6 weeks post-lesion (Fig. 3b), with statistical significance found between CLP6w and CLP2w (*p* < 0.05).

### Macrophage local delivery promotes IVD hernia regression

To validate macrophages as potential therapeutic agents toward hernia regression, unpolarized macrophages, differentiated from syngenic rat bone marrow precursors, were adoptively transferred into the rat IVD hernia site. The animals were sacrificed at 6 weeks post-lesion (Mac6w). Control groups without any treatment other than the lesion, were sacrificed at 2 (L2w) and 6 (L6w) weeks post-lesion (Fig. 1b). Detailed local and systemic analyses were performed to assess the therapeutic potential of macrophage administration for in vivo hernia regression.

**Fig. 4:**
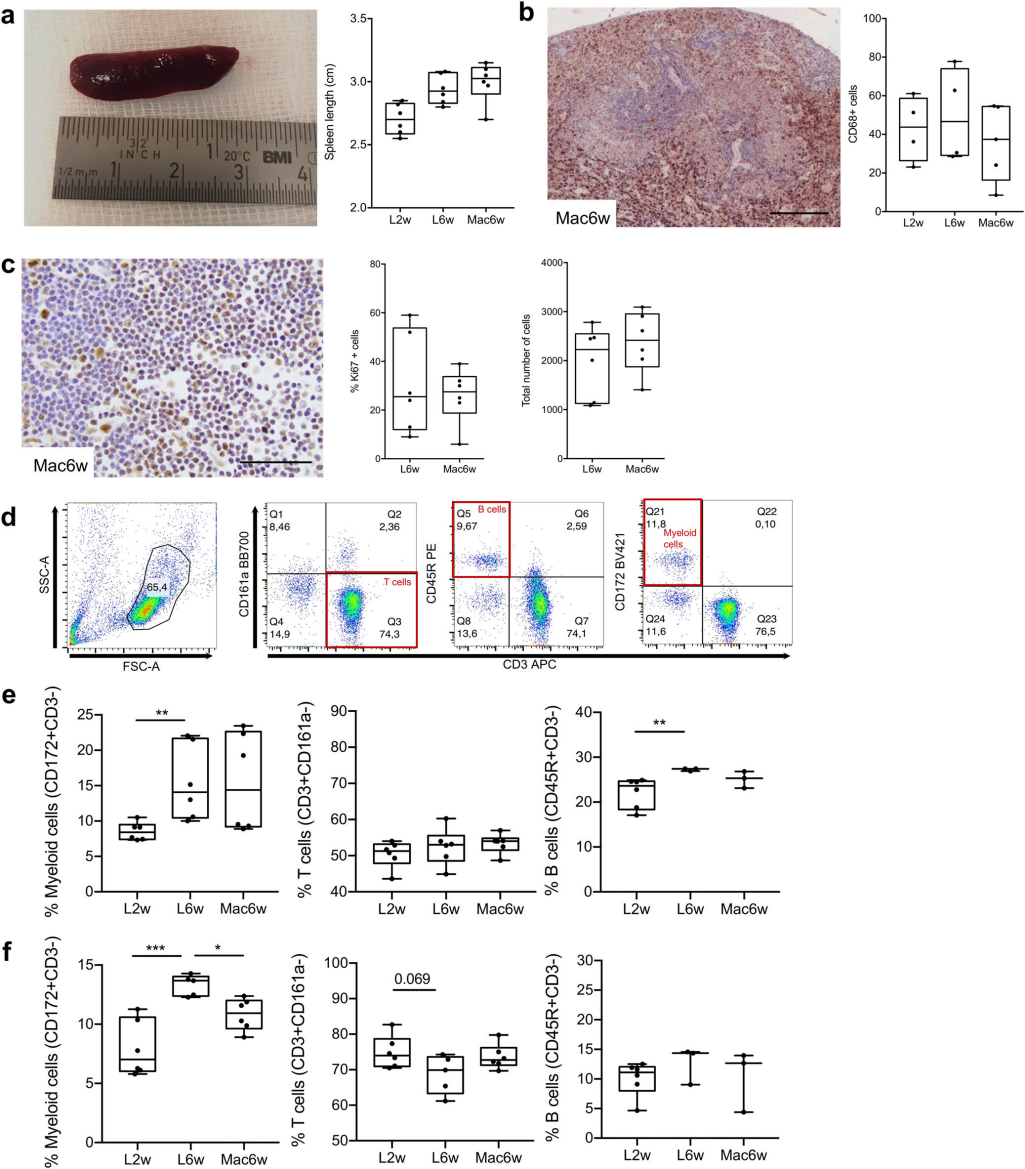
Systemic immune response to macrophage administration into the herniated IVD. **a** Quantification of spleen length. **b** Immunohistochemistry for CD68+ cells in the spleen. Scale bar: 200 µm. **c** Immunohistochemistry for Ki67+ proliferating cells and the total number of cells in the lymph nodes. Scale bar: 50 µm. **d** Representative plots of the flow cytometry gating strategy to identify T-, B-, and myeloid cell populations in peripheral blood and spleen. **e** Spleen cells immunophenotyping by flow cytometry. **f** Peripheral blood mononuclear cells immunophenotyping by flow cytometry. Results are presented as median ± interquartile range (IQR) in box and whiskers plots, and statistical analysis used the non-parametric Kruskal-Wallis test, followed by Dunn’s multiple comparison test, except for (**c**) in which the Mann–Whitney test was used. *p < 0.05, **p < 0.01, ***p < 0.001.

The systemic effect of macrophage administration on the hernia was analyzed in the blood and spleen. There was an enlargement of the spleen in the group administered with macrophages at 6 weeks post-lesion, when compared with the control group 2 weeks post-lesion, but this difference is not significant when compared with the control group 6 weeks post-lesion (Fig. 4a). Immunohistochemistry analysis of the spleen seems to indicate a decrease in CD68+ macrophages in the Mac6w group, although not statistically significant (Fig. 4b). No alterations were found in proliferating or total number of cells in the lymph nodes between L6w and Mac6w groups (Fig. 4c). A detailed analysis of the proportions of different immune cell populations by flow cytometry (Fig. 4d) showed a significant increase in myeloid and B cell proportions at 6 weeks, when compared to 2 weeks post-lesion, but did not reveal any significant alterations in the proportion of immune cell populations associated to macrophage administration (Fig. 4e). Flow cytometry analysis in the blood showed a significant reduction (*p* < 0.05) in the proportion of CD172+ myeloid cells in the blood, in the macrophage-administered group, without significant alterations in the proportion of T cells or B cells (Fig. 4f).

**Fig. 5:**
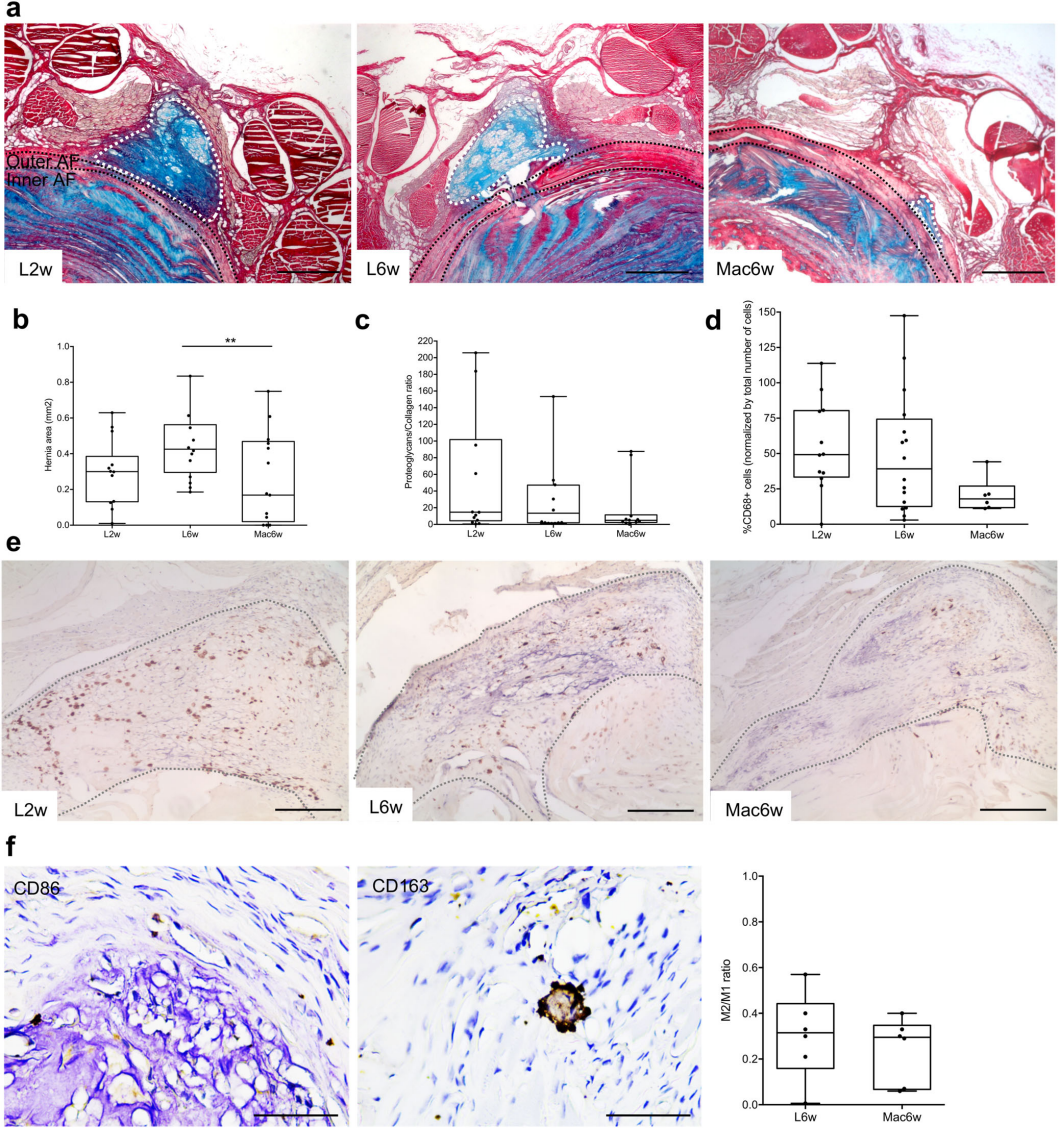
Hernia histopathological analysis at 2 and 6 weeks post-injury and after macrophage administration. **a** Representative images of the hernia stained by Alcian blue (proteoglycans) and Picrosirius red (collagen). White dashed lines outline the hernia. Black dashed lines delineate the outer and inner annulus fibrosus (AF). Scale bars: 500 µm. **b** Quantification of average hernia area (mm^2^) across the depth of all sections of each IVD with visible herniation. **c** Proteoglycans/collagen content ratio in the hernia. **d** Quantification of CD68+ cells within the hernia. **e** Representative images of CD68+ cells within the hernia (positive cells are shown in brown). The dashed line outlines the hernia. Scale bars: 200 µm. **f** Quantification of M1 (CD86+) and M2 (CD163+) macrophage within the hernia (positive cells are shown in brown). Scale bars: 50 µm. Results are presented as median ± interquartile range (IQR) in box and whiskers plots, and statistical analysis used the non-parametric Kruskal–Wallis test, followed by Dunn’s multiple comparison test, except for (**e**) in which the Mann–Whitney test was used. **p < 0.01.

At the local IVD level, the herniated area was delimited by histological analysis of proteoglycan/collagen staining (Fig. 5a). Results revealed a statistically significant 44% decrease in the average hernia size in the group in which macrophages were administered (Fig. 5b). In this group there was also a slight decrease in the proteoglycans/collagen ratio within the hernia (Fig. 5c). Also, we quantified the number of CD68+ macrophages present in the hernia and consistently found fewer macrophages in the macrophage-administered group, albeit the differences were not statistically significant (Fig. 5d). Furthermore, quantification of the M1 (CD86) and M2 (CD163) macrophage subsets indicate a predominance of M1 macrophages within the hernia, with no statistical difference in the M2/M1 ratio between the groups L6w and Mac6w (Fig. 5f).

### Systemic proteomic and cytokine profile unveils a putative macrophage-induced hernia regression mechanism

**Fig. 6:**
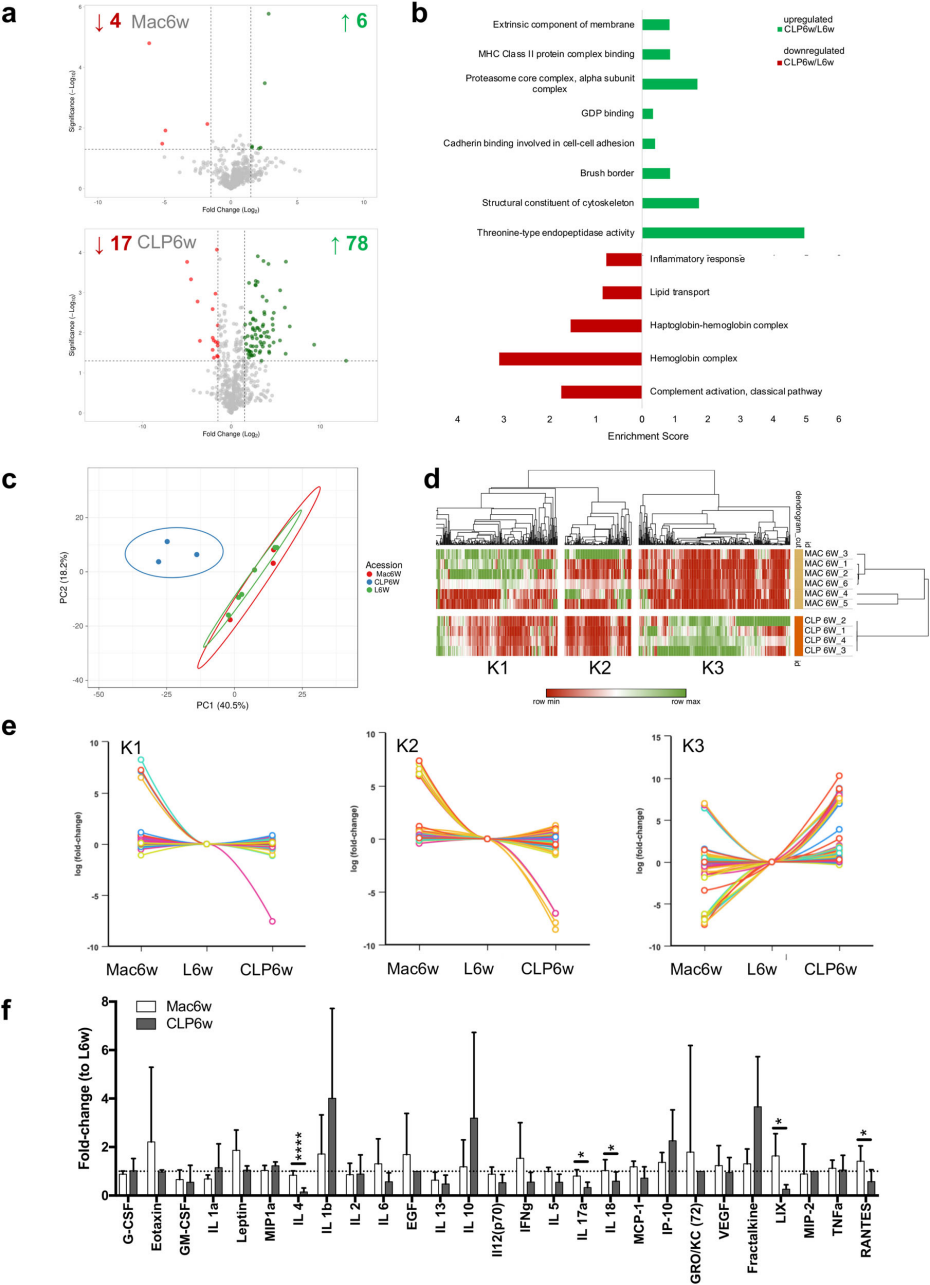
Plasma profile of herniated IVDs, upon macrophage administration and depletion. **a** Volcano plot of down and upregulated proteins found in the Mac6w and CLP6w groups, compared to L6w. **b** Gene Ontology terms (cellular component, molecular function, and biological process) found in the upregulated and downregulated proteins in CLP6w, with respect to L6w. Cut-off > 1.5, *p* < 0.05. **c** Principal Component Analysis of Mac6w, CLP6w and L6w groups, showing a clear separation in the CLP6w group, from the Mac6w and L6w groups. This is further evidenced in the Heatmap (**d**), where three protein clusters were distinguished (**e**). **f** Plasma cytokine/chemokine profile normalized to L6w levels (dashed line at *y* = 1). Results are presented as median ± interquartile range (IQR) in box and whiskers plots, and statistical analysis used the non-parametric Kruskal–Wallis test, followed by Dunn’s multiple comparison test. *p < 0.05, ****p < 0.0001.

To further explore the systemic response to the hernia in macrophage-administered and depleted conditions, proteomic analysis by LC–MS/MS was performed in the plasma of animals from Mac6w and CLP6w groups, as well as from their respective control groups (L6w). Differential analysis using a Volcano plot showed that, among the 631 proteins identified in the Mac6w group, only 6 were found significantly upregulated and 4 significantly downregulated when compared to the lesion. In contrast, a total of 640 proteins were identified in the CLP6w group, with 78 proteins being significantly upregulated and 17 significantly downregulated (Fig. 6a). These results indicate that, as expected, the majority of the proteins found in the plasma were unaltered by local macrophage administration, as opposed to the systemic depletion. To explore the biological processes affected by the differentially expressed proteins (cut-off > 1.5, *p* < 0.05), Gene Ontology (GO) term enrichment analysis was performed. Given the reduced number of differentially expressed proteins in the Mac6w group, no GO terms enrichment was identified. For the CLP6w group, the upregulated proteins were enriched in GO terms associated with “cadherin binding involved in cell-cell adhesion”, “brush border”, “structural constituent of cytoskeleton”, “threonine-type endopeptidase activity”, and “MHC class II protein complex binding”, among others. This is in accordance with the flow cytometry results since MHC class II regulates B cells (among others), and an overall increase in its proportion was observed in both blood and spleen at 6 weeks^19^. On the other hand, the downregulated proteins returned GO terms related to “complement activation”, “inflammatory response”, and “hemoglobin complex” (Fig. 6b), indicating downregulation of inflammation, as anticipated in a macrophage depletion scenario.

Moreover, principal component analysis (PCA) (Fig. 6c) showed a clear distinction between the CLP6w group and both the L6w and Mac6w groups, once more confirming the reduced systemic effects associated with macrophage administration, unlike its depletion, which clearly resulted in a strong systemic response. Furthermore, a heat map was produced (Fig. 6d), where three clusters were selected and analyzed for GO terms (Fig. 6e). In the first cluster (K1), protein expression appears to decrease in the CLP6w group, compared to Mac6w, and the GO terms search resulted in “complement activation”, “phagocytosis and engulfment”, “actin filament capping”, “podosome”, “collagen trimer” “positive regulation of insulin-like growth factor (IGF) signaling pathway”, “regulation of cell growth”, “negative regulation of cell proliferation”, “positive regulation of MAPK cascade”, “positive regulation of cell migration”, “positive regulation of ERK1 and ERK2”. Overall, these GO terms seem to refer to the improvement of macrophage phagocytic activity, tissue remodeling, and associated pathways upon therapy application. For the K2, where proteins once again seem downregulated in the CLP6w group, the GO terms “cellular response to oxidative stress (MAPK cascade)” and “cellular response to reactive oxygen species” were found, which is in accordance with the previous cluster. Finally, in K3, proteins seem to be up-regulated in the CLP6w group, and the associated GO terms were “focal adhesion”, “intermediate filaments”, “structural constituent of cytoskeleton”, “actin filament”, “positive regulation of vasoconstriction”, “mitotic cell cycle” and “ATPase activity”, which may be related to macrophage depletion itself.

To further explore the mechanism behind the potential increase in macrophage phagocytic activity unveiled by proteomics, a panel of 27 systemic inflammatory mediators was analyzed in the plasma by cytokine array (Fig. 6f). Results show that IL4, IL17a, IL18, LIX, and RANTES were significantly increased in the Mac6w group, compared to CLP6w. Overall, these results seem to indicate a possible mechanism for macrophage-induced hernia regression via macrophage activation and phagocytic capacity through IL4, IL17a, IL18, and RANTES and intensified tissue remodeling mediated by LIX.

## Discussion

This study clearly shows the important role of macrophages in hernia regression and represents the first preclinical proof-of-concept of macrophage therapy for IVD hernia regression. Clodronate-mediated macrophage depletion increased hernia sizes in a rat IVD herniation model. Conversely, bone marrow-derived macrophages were administered into the herniated IVD, and a statistically significant 44% decrease in the hernia sizes was obtained. Overall, the results presented demonstrate the central role of macrophages in IVD hernia regression and its potential as a valid therapeutic approach. A possible mechanism of action is also unraveled.

For most lumbar IVD herniations, the gold standard surgical approach is microdiscectomy^20^. Minimally invasive therapies have gained a high momentum to address the spine, as they minimize the risk of infection and post-surgical complications. However, there are still complications associated, such as neural injuries and cerebrospinal fluid leaks. Cell therapies to halt, delay or repair the degenerated IVD have been studied for a long time and show good promise, reviewed in^21,22^. In particular, bone marrow-derived MSCs, adipose tissue-derived MSCs, and disc-derived chondrocytes have been or are presently under clinical trials reviewed in^23^. However, there are still several challenges to overcome, including a lack of standardization in preclinical testing, an absence of guidelines in patient stratification, and an incomplete understanding of the biological mechanisms^21^. Nonetheless, cell transplantation in the IVD is technically feasible, and percutaneous delivery is possible. Previous work in a dog model of IVD degeneration demonstrated that transplanted disc chondrocytes were able to integrate with the surrounding tissue, produce the appropriate IVD ECM and potentially provide a functional solution to repair the IVD^24^. Such strategies intend to provide a biological restoration of the degenerated or lost IVD tissue. Conversely, our strategy takes advantage of the natural physiological mechanisms of spontaneous hernia resorption, potentiating it by administering macrophages, which we demonstrate to have a central role in hernia regression. This concept is new to clinical practice and goes beyond the current exogenous cell/gene/biomaterial administration strategies used in tissue engineering. We have used bone marrow-derived macrophages for in situ administration. These cells are recruited during inflammation and typically exhibit distinct transcriptional, functional, and phenotypic signatures from resident macrophages^25^. However, macrophages are plastic cells, highly specialized and able to polarize towards specific functional phenotypes according to the microenvironment^26^. This study demonstrates that exogenously administered bone marrow-derived macrophages are able to have a positive biological effect on the hernia, despite the harsh IVD environment, with low irrigation and high mechanical stress, which challenges cell administration and survival.

Transplantation of immune cells, particularly macrophages, bears the risk of immune rejection. In the clinical setting, autologous macrophages will be used, but for the scope of this study, we have used an inbred rat strain (LEW/Crl) and macrophages isolated from the bone marrow of syngeneic rats. In this way, potential immune rejection was minimized. Also, we have adopted a minimally invasive percutaneous administration route, which is the gold standard for cell therapy clinical trials targeting IVD. To address safety concerns, we have conducted a thorough analysis of possible systemic effects resulting from macrophage IVD hernia administration. The results were very positive since no side effects were observed locally or systemically in the blood, plasma, or spleen.

More than a demonstration of the functional role of macrophages in IVD hernia regression, this study further represents a new immunotherapeutic approach for IVD hernia regression. In the last decade, immunotherapy has been regarded as a major breakthrough in the field of anti-cancer therapy. It is now clear that macrophages contribute significantly to the final outcome of different cancer immunotherapy strategies^27^, and they are already a promising target itself in cancer immunotherapies. CAR-M cells have already been proposed by modifying macrophages with CAR technology to target solid tumors^28^. Outside of the cancer immunotherapy field, they have only now started to be explored as a therapy. Macrophage transplantation was proposed to improve skeletal muscle function after injury^29^ and recently for myocardial repair^30^. Immunotherapy to target IVD hernia is completely novel. Furthermore, the possibility exists to further engineer macrophages^31^ to target IVD hernia. The concept of immunotherapy for tissue engineering strategies is still underexplored but has the potential to provide important breakthroughs to currently unsolved clinical problems.

A central concern for the clinical translation of such therapy regards the macrophage role in the inherent inflammatory milieu of an IVD hernia. This is because, despite the role of the macrophage in homeostatic phagocytosis, macrophage-driven inflammation may also contribute to the pathophysiology of IVD herniation reviewed in^5^. Indeed, recent reports show that increased infiltration of macrophages in the dorsal root ganglia is associated with the initiation and maintenance of neuropathic pain^32^ and lumbar disc herniation-induced sciatica^33^. On the other hand, in a recent systematic study that highlights the contradictory effect of macrophage-related cytokine expression in lumbar disc herniations, it was found that the presence of macrophages was negatively associated with pain scores^34^. Further studies in this area will be needed, but the challenge for clinical translation of any macrophage-based therapy will be to understand the molecular pathways regulating macrophage function during IVD herniation for controlling and properly balancing its inflammatory status.

More than pre-clinical proof of concept of macrophage-based immunotherapy for IVD herniation, this study also unveils a putative mechanism through IL4, IL17a, IL18, and RANTES-mediated macrophage phagocytic activity and an increased tissue remodeling possibly promoted by LIX. Indeed, IL4, IL17a, and IL18 are pro-inflammatory cytokines, and the first two are commonly associated with M2 macrophage activation. M2 macrophages exhibit a potent phagocytic capacity to scavenge debris and apoptotic cells, being responsible for moderating inflammation and promoting tissue repairing^35,36^. RANTES has also been described as an important modulator of macrophage functions, promoting its aggregation, chemotaxis, and phagocytosis^37^. Finally, LIX is an important cytokine in neutrophil recruitment^38^ and has been reported as having a role in connective tissue remodeling^39^. Moreover, the proteomic analysis also identified the IGF signaling pathway as associated with macrophage activity role in hernia regression. Indeed, macrophages are known to release IGF-1 during apoptotic cell engulfment or in response to inflammatory cytokines such as IL4^40^. Moreover, at the onset of IVD degeneration, its activation increases cell proliferation and promotes ECM synthesis while inhibiting its decomposition and preventing disc cell senescence^41^.

Clearly, a series of detailed analyses will be further needed to integrate future successful clinical translation of this therapy. These include profiling the most effective macrophage phenotypes to be administered, optimizing delivery time, route, and dosage, further investigating the safety, and also validating results with human macrophages. Moreover, to limit the number of variables, including sex hormones, in this first proof-of-concept of macrophage therapeutic potential for IVD hernia regression, the current study used only male animals. Thus, other studies using female and also older animals should be conducted to validate the results presented here. Moreover, other complementary animal models of IVD herniation, including large animal models, should be considered since the rat IVD, unlike human IVD, contains abundant notochordal cell populations and gelatinous NPs until late in its maturation^42^. Finally, assessing whether macrophage therapy results in the reduction of IVD herniation-associated pain using adequate models of quantitative sensory testing (e.g.,^43^) will be one of the important questions in clinical translation.

The envisaged clinical therapy consists in a minimally invasive autologous adoptive cell transfer, in which monocytes are isolated from the patient’s peripheral blood, polarized in vitro, and returned to the same patient in a similar way to what is already performed for dendritic cells and T-cells cancer immunotherapies. Understanding which IVD hernia patients will benefit the most from this therapeutic approach will be key to a successful personalized treatment.

This study represents the first preclinical proof-of-concept of macrophage-based therapy for IVD hernia regression. This immunotherapy relies on the potentiation of the physiological hernia regression mechanisms. Further development of this macrophage-based therapy for clinical application is warranted.

## Methods

### IVD lesion and herniation model

Male Lewis (LEW/Crl) rats at 2–3 months of age were used for the IVD caudal herniation model, as previously described^7^. This inbred strain was used to reduce possible allogeneic cell rejection. Briefly, the animals were anesthetized by isoflurane inhalation, placed in a prone position, and the tail skin was disinfected with ethanol. A percutaneous 21-G needle puncture was performed in the coccygeal IVDs Co5/6, Co6/7, and Co7/8, using radiography for IVD identification. Experiments were carried out at i3S—Instituto de Investigação e Inovação em Saúde animal facility and were approved by the i3S Animal Welfare and Ethics Review Body and the Portuguese Competent Authority (DGAV) (license n° 3773/2015–02–09) and conducted in accordance with the European Legislation on Animal Experimentation through Directive 2010/63/UE. For the first experimental setup, multiple CLP injections were administered to one group of animals between the lesion and 2 weeks post-lesion (CLP2w, *n* = 6) and to another group of animals between 2 and 6 weeks post-lesion (CLP6w, *n* = 5). Control groups consisted of lesioned animals sacrificed at 2w (L2w, *n* = 3) and 6w (L6w, *n* = 3) post-lesion, without CLPs administration (Fig. 1a). For the second experimental setup, one group of animals received macrophage administration at 2 weeks post-lesion and was sacrificed at 6 weeks post-lesion (Group Mac6w, *n* = 6). Control groups consisted in lesioned animals sacrificed at 2w (L2w, *n* = 6) and 6w (L6w, *n* = 6) post-lesion, without macrophage administration (Fig. 1b).

### Macrophage systemic depletion via administration of CLPs

Commercially available liposomes loaded with clodronate (dichloromethylene-bisphosphonate, Liposoma) were prepared according to the manufacturer’s instructions. Briefly, the solution was allowed to reach room temperature and then gently shaken. CLP was administered at a dosage of 100 µl per 10 g of body weight via intravenous injection in the lateral tail vein using a 24-G catheter (Braun) under general anesthesia. Group CLP2w received CLP immediately after IVD lesion and then repeated administrations every 3 days until 2 weeks post-lesion. Group CLP6w received CLP starting from 2 weeks post-lesion and then repeated administrations every 5 days until 6 weeks post-lesion.

### Macrophage isolation and administration into the IVD hernia

Macrophages were isolated from the bone marrow of syngeneic rats. Tibial and femur bone marrow were collected by flushing, as previously performed for isolation of MSCs^8^. Isolated cells were cultured in RPMI + 10% FBS media, supplemented with rrM-CSF for 7 days, for macrophage differentiation. Unpolarized adherent and non-adherent cells were collected using Accutase, and cells were resuspended in a sterile saline solution. Each administration consisted of 1 × 10^6^ cells resuspended in 10 µl of sterile saline solution injected locally in the hernia site 2 weeks after IVD lesion, using a 31-G needle coupled to a microsyringe (Hamilton) with an adapter to ensure 2.5 mm depth administration to the hernia site. All cell culture was performed at 37 °C in an atmosphere of 5% CO_2_, and cell handling procedures were performed in a sterile laminar flow hood.

### Tissue collection

Total blood was collected by intracardiac puncture, under isoflurane anesthesia, into an anti-coagulant EDTA solution. Animals were then dissected for collection of the spleen and the lymph nodes. The spleen was photographed, and half was used for histology, while the other half was immediately processed for flow cytometry analysis. The lymph nodes were processed for histology. Blood was centrifuged at 800*g* for 20 min at room temperature, and then plasma and buffy coats were separately collected. Plasma was further centrifuged twice at 2500*g* for 15 min at 4 °C to remove cell debris and kept at −80 °C for cytokine array and proteomic analysis. Collected buffy coats were diluted with PBS, overlaid on Lymphoprep in a 1:1 ratio, and centrifuged at 800*g*, for 30 min at room temperature, without brake, to isolate peripheral blood mononuclear cells (PBMC), which were immediately analyzed by flow cytometry. Spleen cells were isolated by mechanical and enzymatic dissociation, using 100 U/mL Collagenase type I (Sigma) and a 100 μm pore cell strainer. Red blood cells in spleen cell suspension were further lysed by incubation with NH_4_Cl 150 mM in Tris 10 mM solution for 8 min at 37 °C. The other half of the spleen was fixated in 10% neutral buffered formalin and processed for paraffin embedding. Sections of 3 µm were obtained and stained for immunohistochemistry (CD68).

### IVD histology and quantification of hernia area

Each functional spinal unit (one IVD and two adjacent vertebrae) was collected *en bloc* and fixated in 10% neutral buffered formalin for 1 week at room temperature. Tissue was decalcified in EDTA-glycerol solution and processed for paraffin embedding. Sequential transversal 5 µm sections of the IVD were collected. Sections were either stained with Alcian blue/Picrosirius red (AB/PSR), with Hematoxylin and Eosin, or used for immunohistochemistry. Briefly, for the AB/PSR staining, sections were deparaffinized, rehydrated, incubated in Weigert’s Iron Hematoxylin for 10 min, washed in tap water, and stained in Alcian blue solution pH 2.5 for 30 min. After rinsing in tap water, sections were incubated in Picrosirius red solution (0.1 g Sirius red in 100 mL of saturated aqueous picric acid solution) for 1 h, followed by washing in 0.01 N HCl for 2 min. Sections were dehydrated, mounted with Entellan (Merck), and analyzed in a CX31 optical microscope equipped with a DP25 digital color camera (Olympus). The herniated area was determined by delimitating regions of interest (ROI) in each optical section, using the freehand selection tool in the ImageJ software, considering blue staining for proteoglycans and red staining for collagen. The mean herniated area for each animal was calculated as the mean of areas of each individual section throughout the IVD, as described before^7^. Within ROIs, the proteoglycans and collagen percentage of the area were determined by a custom ImageJ macro based on a color deconvolution technique used to separate the different color channels^44^.

### Immunohistochemistry

Immunohistochemistry for the detection of CD68+ cells in the hernia was performed by the Novolink^TM^ Polymer Detection Kit (Leica Biosystems), following the manufacturer’s instructions. Antigen retrieval was performed through incubation in a near-boiling point of 10 mmol/L sodium citrate buffer, pH 6.0, for 1 min. Sections were incubated with anti-CD68 antibody (clone ED1, 1:100 dilution, Bio-Rad Laboratories) overnight at 4 °C. Immunohistochemistry for detection of CD86+ and CD163+ cells in the hernia was performed by the Poliview Plus-HRP (Anti-mouse) (Enzo) for 30 min and the reaction was developed with DAB Quanto (Epredia) for 2 min. Antigen retrieval was performed through incubation in a near-boiling point 10 mmol/L sodium citrate buffer, pH 6.0, for 1.5 min. For CD163, further digestion was performed with proteinase K for 6 min at RT. Sections were incubated with anti-CD86 antibody (1:300 dilution, Bio-Rad Laboratories) and anti-CD163 antibody (1:25, Bio-Rad Laboratories) overnight at 4 °C. Stained sections were imaged with light microscopy (Axiovert 200 M, Zeiss). Positive cells were quantified on the acquired images using the Cell Counter plugin of ImageJ software. For CD68, the total number of positive cells was divided by the total number of cells in the hernia. For the M2/M1 ratio, the total number of CD163 positive cells was divided by the total number of CD86 positive cells in the hernia.

### Flow cytometry analysis

Flow cytometry analysis was performed for main immune cell populations, namely myeloid and lymphocytic (T, B, and NK cells) lineages, with panels of fluorochrome-conjugated antibodies for cell surface lineage and activation markers. Staining was performed in 96-well plates in FACS buffer (0.5% BSA, 0.01% sodium azide, PBS) for 30 min on ice after Fc receptors blocking. The following anti-rat antibodies were used: Mix1: CD161a(clone 10/78)-BB700, CD172(clone OX-41)-BV421, CD3(clone IF4)-APC, CD45R(clone HIS24)-PE, CD11b/c(clone OX-42)-PE-Cy7, major histocompatibility complex class II (MHCII)(clone OX-6)-BV510, CD8a(clone OX-8)-FITC, CD4(clone OX-35)-APC-Cy7; Mix2: CD172(clone OX-41)-BV421, CD86(clone 24F)-PE, CD11b/c(clone OX-42)-PE-Cy7, CD40(clone HM40–3)-FITC, major histocompatibility complex class II (MHCII)(clone OX-6)-PerCP, CD163(clone ED2)-DyLight®650. CD163 was previously conjugated with DyLight®650 using the LYNX Rapid Plus Antibody Conjugation Kit, according to the manufacturer’s instructions. All antibodies were from BD, except CD163 and the conjugation kit, which were from BioRad. Cells were further incubated with the fixable viability dye eFluor®780 (Invitrogen). Samples were acquired on a flow cytometer (FACSCanto II; BD), and obtained data were analyzed with FlowJo software version 8.7 (FlowJo, Ashland).

### Plasma cytokine array

Quantitative analysis of 27 cytokines and growth factors in the plasma was performed using the commercial Rat Cytokine Array/Chemokine Array 27-Plex Discovery Assay (Eve Technologies Corp.). Plasma samples were diluted in PBS to a dilution factor of 2, according to the manufacturer’s instructions. All measurements were performed by Eve Technologies Corp.

### Plasma proteomic analysis

Plasma was centrifuged at 15000 g for 15 min at 4 °C, total protein was quantified by the BCA method, and 100 µg of total protein was solubilized and processed for proteomic analysis following the solid-phase-enhanced sample-preparation (SP3) protocol as described in^45^. After enzymatic digestion, protein identification and quantitation were performed by label-free high-resolution accurate-mass nanoLC-MS/MS, which is composed of an Ultimate 3000 liquid chromatography system coupled to a Q-Exactive Hybrid Quadrupole-Orbitrap mass spectrometer (Thermo Scientific), as described^46^. Data acquisition was controlled by Xcalibur 4.0 and Tune 2.11 software (Thermo Scientific). Raw data were processed using Proteome Discoverer 2.5.0.400 software (Thermo Scientific) and searched against the UniProt database for the *Rattus norvegicus* Proteome 2020_05 and a predicted spectral library. A common protein contaminant list from MaxQuant was also considered. The MSPepSearch and Sequest HT search engines were used to identify tryptic peptides. Data were filtered by employing an FDR threshold of 1% and considering only unique or razor peptides higher than 2. Contaminants were removed. Volcano plots were created with VolcaNoseR software, PCA with ClustVis software, and the heatmap was generated with Morpheus software. Gene Ontology (GO) term enrichment was assessed using the Functional Annotation Clustering Tool from DAVID Database, as previously performed^47^.

### Statistical analysis

Normality was assessed by the D’Agostino-Pearson omnibus normality test. Results are presented as median ± interquartile range in box and whiskers plots. Statistical analysis was performed with the non-parametric Mann–Whitney test whenever two groups were compared and with the Kruskal–Wallis test, followed by Dunn’s multiple comparison test, whenever more than two groups were compared. GraphPad Prism 7 software was used. Statistical significance was set at *p* < 0.05.

### Reporting summary

Further information on research design is available in the Nature Research Reporting Summary linked to this article.

## Supplementary information

Reporting Summary

## Data Availability

The data that support the findings of this study are available from the corresponding author upon reasonable request. The proteomics data have been deposited to the ProteomeXchange Consortium via the PRIDE^48^ partner repository with the dataset identifier PXD042802.
